# Biocompatibility and Antimicrobial Activity of Nanostructured Lipid Carriers for Topical Applications Are Affected by Type of Oils Used in Their Composition

**DOI:** 10.3390/pharmaceutics13111950

**Published:** 2021-11-17

**Authors:** Dragana P. C. de Barros, Patricia Reed, Marta Alves, Rafaela Santos, Abel Oliva

**Affiliations:** 1 Instituto de Tecnologia Química e Biológica António Xavier, NOVA University Lisbon, 2780-157 Oeiras, Portugal; preed@itqb.unl.pt (P.R.); marta.alves@itqb.unl.pt (M.A.); rafaela.santos@itqb.unl.pt (R.S.); oliva@itqb.unl.pt (A.O.); 2 Institute of Experimental Biology and Technology, Apartado 12, 2781-901 Oeiras, Portugal

**Keywords:** NLCs, plant oils, cytotoxicity, *S. aureus*, antimicrobial activity

## Abstract

Nanostructured lipid carriers (NLCs) have gained significant attention as tools for the dermal delivery of therapeutics due to their stability, biocompatibility, and ability to improve drug bioavailability. The use of natural plant oils (NPO) in NLC formulations has numerous benefits for the skin due to their therapeutic potential. This work shows the effect of NLC composition on bioavailability in epidermal cells and antimicrobial activity against *Staphylococcus aureus*. Sixteen systems containing fixed (sunflower, olive, corn, peanut, coconut, castor, and sweet almond) and essential (eucalyptus) oils, with different solid lipid (SL): liquid lipid (LL) ratios, were engineered. The structural properties, bioavailability, and antimicrobial action of the particles was studied. The choice of NPO influenced the physicochemical stability by changing the diameter of NLC formulations (between 160 nm and 185 nm) and Z-potential (between −46 mV and −61 mV). All of the systems were characterized by concentration-dependent cytocompatibility with human epidermal keratinocytes (HaCaT) and human dermal fibroblasts (HDFn). The SL:LL ratio in some NLC systems impacted cell cytotoxicity differently. Antimicrobial properties were observed in all 16 systems; however, the type of oil and SL:LL ratio affected the activity of the formulations. Two NLC-NPO systems were found to be non-cytotoxic to human cells lines at concentrations that completely inhibited bacterial growth. These results present a strong argument that the use of natural oils in NLC formulations presents a promising tool for the treatment of skin infections.

## 1. Introduction

The applications of nanoparticles (NPs) and other colloidal drug-delivery systems in cutaneous drug delivery modify the drug distribution and release profile [1]. The dermal absorption of nanoparticles assumes direct contact with the nanostructured material, ensuring close contact with the stratum corneum and increasing the amount of drug absorbed by the skin. Nanostructured lipid carriers (NLCs) have been shown to improve controlled drug release, lower toxicity, and increase the bioavailability of drugs, such as antibiotics [2,3,4,5,6].

NLC comprises a broad spectrum of diverse, biocompatible, and biodegradable lipids that are GRAS (Generally Recognized As Safe) approved [7,8]. The selection and good understanding of physicochemical properties of liquid lipids are some of the most critical factors affecting performance and stability of NLCs [9]. Yang et al. reported that NLC suspension stability was strongly affected by the type and amount of the carrier oil [10]. Pinto et al. recently showed that the particle size, size distribution, and surface charge of the natural oils-based lipid nanoparticles are significantly influenced by the composition of the lipid core [11,12]. The use of natural ingredients, such as plant oils, fixed and essential, in NLC formulations improves the biological activity and advantages of these carriers [13]. Fixed plant oils contain triglycerides, phospholipids, waxes, saturated and unsaturated free fatty acids (FFAs), nutrients, vitamins, minerals, and polyphenolic compounds [14]. Essential oils are natural, plant-derived aromatic, clear, and volatile oily liquids. From a chemical point of view, they are mixtures of low molecular weight terpenes and phenyl compounds, and other volatile components [15,16]. The composition of plant oils, when topically applied, influences skin physiology (skin barrier, inflammatory status, antioxidant response, and proliferation) differently and have been recognized for their beneficial effect on the skin [14,15,16,17]. Additionally, the plant composition of oils (specifically monounsaturated FFAs, polyphenolic compounds) in NLC formulations can act as chemical enhancers, improving drug penetration into the skin, and interacting with the skin lipids to promote various therapeutic effects [18]. Commercialization of natural oils in NLC formulations was mostly for dermal applications [13,19]. For example, Nano Repair Q 10 cream and Serum use sunflower seed extract for the liquid phase [19]. There is far less data about NLC safety than efficiency, as these systems are often considered automatically safe for cosmetic purposes using GRAS ingredients. However, the application of marketing authorization for pharmaceutical products requires detailed data on efficacy, quality, and safety. In vitro toxicity of nanoparticles in cultured cells is the first step to determine the biocompatibility of nanomaterials on living cells [20].

The antimicrobial effects of lipid-based particles such as liposomes, solid lipid nanoparticles and, recently, NLCs have been reported [21]. Cutaneous colonization models demonstrated that *Staphylococcus aureus*, part of the normal flora in approximately 30% of people yet can be a tenacious pathogen [22], is the leading cause (80–90%) of skin and soft tissue infections (SSTIs) [23,24]. The bacteria are facilely located in wounded skin, playing a critical role in infection-induced inflammation and cutaneous disease progression [25]. Apart from the intracellular survival behavior of *S. aureus*, the lack of efficient penetration of antibiotics across the mammalian cell membrane also leads to difficulty in the treatment [26]. One of the strategies to increase the accumulation of antibiotics in cells is developing antibiotic nanoparticle drug delivery systems [4,5,27]. Additionally, antimicrobial resistance (AMR) is considered one of the greatest threats to human health [28]. The antibiotic discovery pipeline is virtually dry, and current antibiotic treatment is often ineffective. The antibacterial action of substances from natural origins incorporated into NLCs has been extensively studied over the past few years [29,30,31]. Encapsulation can be a suitable strategy to protect antimicrobial substances against some harsh conditions of processing and storage and to provide efficient formulations for antimicrobials [21]. Therefore, the development of alternative drug administration approaches and novel antimicrobials is imperative. Natural oil-based NLC formulations have improved biological activity and have shown antimicrobial effects against human skin pathogens [21,30,31]. However, most research showed the synergic efficacy of NLC–drug conjugate systems [21,30,31,32,33,34,35,36]. Therefore, to analyze the antimicrobial efficacy of natural compounds integrated in NLC formulations, empty NLC-NPO systems with different oils should be tested for biocompatibility and antimicrobial activity.

In this study, to assess the role of NLC composition (with the focus on FFAs) in terms of cytotoxicity, we investigated cell viability in HaCaT and HDF cells incubated with eight different blank NLC formulations differing in their liquid lipid composition. Amongst various therapeutic potentials of FFAs, the antibacterial properties were already reported [37]. We chose plant oils based on different saturated vs. unsaturated FFA compositions and their proven benefits for application to the skin. Seven fixed oils (sunflower (SF), olive (OV), corn (CO), peanut (PO), coconut (CC), castor (CS), sweet almond (SA)) were chosen as lipid liquids. Additionally, we tested a eucalyptus oil based NLC, as this essential oil has been shown to have a very strong antimicrobial activity and a very low-fat content [38,39,40]; however, it can provoke dermatitis reactions on the skin in its free form. The surfactant composition of the NLCs was optimized, and physicochemical characterization with crystallinity studies of the lipid matrix was performed for all systems. The connection between cytotoxic and antimicrobial concentrations for explored systems and the internalization of lipid carriers in epidermal cells will be discussed.

## 2. Materials and Methods

### 2.1. Materials

Solid lipid: Myristic acid, C14:0 (98%) was purchased from Alfa Aesar (Haverhill, MA, EUA). Liquid lipid: Sunflower (SF) oil, corn oil (CO), peanut oil (PO), (Fula, Portugal), and olive oil (OV) (Gallo, Portugal) were food grade commercial products. Sweet almond (SA) oil from *Prunus Amygdalus Dulcis*, castor oil from *Ricinus communis* (F.J. Campos, Portugal), and virgin coconut oil (CC) (Fauser Vitaquell, Hamburg, Germany) were cosmetic-grade products produced by cold pressure. Eucalyptus oil (EO) from *Eucalyptus globulus* 100% pure was purchased from Biover, France. Ratios of saturated/unsaturated fats in oils are given in Table 1. Span 80 (Sorbitan monooleate, HLB 4.7) was bought from Alfa Aesar (Haverhill, MA, EUA). The aqueous phase of mini-emulsions was prepared using Milli-Q grade water. Cell lines: Human immortalized keratinocytes (HaCaT) and human dermal fibroblasts, neonatal (HDFn), the cell media reagents, DMEM (Dulbecco’s modified Eagle’s medium), fetal bovine serum (FBS), trypsin 0.25%, Pen Strep (10,000 U/mL penicillin, 10 µg/mL streptomycin), Trypsin-EDTA (0.25%), phenol red, phosphate-buffered saline (PBS) 1X, pH 7.4, and the reagent MTT (3-(4,5-dimethylthiazol-2-yl)-2,5-diphenyltetrazolium bromide) were purchased from Gibco, ThermoFisher Scientific (Waltham, MA, USA). The fluorophore for NLC staining, ‘DiO’, DiOC18(3) (3,3′-dioctadecyloxacarbocyanine perchlorate) was bought from Marker Gene Technologies, Inc. (Eugene, OR, USA). Cell stain 4′,6-diamidine-2′-phenylindole dihydrochloride (DAPI) was purchased from Bertin (BioReagent, Montigny le Bretonneux, France) and Wheat Germ Agglutinin Conjugates (WGA, Alexa Fluor 594 conjugate) from Invitrogen ThermoFisher Scientific (Waltham, MA, USA). Formalin solution (neutral solution (neutral buffered 10%) was obtained from Bio-Optica (Milano, Italy).

**Table 1:** Ratio of saturated and unsaturated fats in oils.

FFAs	Saturated, %	Monosaturated, %	Polysaturated, %
Sunflower oil ^1^	10	28	53
Olive oil ^1^	15	68	8
Corn oil ^1^	13	28	50
Peanut oil ^1^	16	61	15
Coconut oil ^1^	95	2	3
Castor oil ^2^	2	91	7
Sweet almond oil ^2^	11	70	19
Eucalyptus oil ^2^	43	35	19

^1^ Data were taken from supplier; ^2^ Data were taken from literature (see Table 2).

### 2.2. NLC-NPOs Preparation

Seven fixed oils (SF, OV, CO, PO, CC, CS, and SA) and eucalyptus, as an essential oil (EO), were selected for the liquid lipids in the NLC formulations. NLCs were prepared by the mini-emulsions methodology with two different solid lipid (SL): liquid lipid (LL) molar ratios (Table 3), with an ultrasonication step [12]. The aqueous phase was made of the Span 80 solution in Milli-Q water heated to lipid phase temperature. The lipid phase consisted of a blend of a myristic acid (C14:0) as a solid lipid with the selected fixed oil; it was heated to 80 °C, which was above the melting point of the solid lipid used, in order to prevent lipid memory effect [11,12]. Thus, the lipid and aqueous phases were mixed. The pre-mini emulsion was stirred for 2 h at 300 rpm and then fully homogenized with a Sonifier (Branson 450D, Danbury, CT, USA) for 10 min (10 s on/5 s off, 55% amplitude). The resultant nano-emulsion was then cooled to room temperature and stored. Each NLC formulation was prepared and tested in triplicate.

### 2.3. Dynamic Light Scattering (DLS) for Physicochemical Characterization of NLC-NPOs

The NLC-NPOs size and polydispersion index (PDI) were measured by DLS (Malvern Zetasizer Nano ZS (Malvern Instruments, Malvern, UK) at 25 °C under a scattering angle of 173◦ at a wavelength of 633 nm, 24 h after preparation. The samples were added to a cuvette without dilution before the measurement. Particle sizes and PDIs are given as the average of three measurements. The Z-average diameter is related to the intensity of scattered light. The homogeneity of the sample is expressed by the PDI value, which measures the particle size distribution. The equipment’s controlling software performed data processing, and the particle size data were evaluated using the intensity distribution. The NLC-NPOs surface charge expressed as a zeta potential (ZP) was determined by measuring the electrophoretic mobility of the nanoparticles in an electric field using the same equipment. Before measurements, the samples were diluted with Milli-Q water (1:10, *v*/*v*). The reported values are the mean ± standard deviation (SD) of at least three different batches of each NLC formulation. Statistical analysis of variance for particle size, PDI, and ZP was performed with Microsoft Excel 2013 software (Microsoft, Redmond, WA, USA) by normal distribution using a significance level of α = 0.05.

### 2.4. Morphologic and Structural Analysis

Transmission electron microscopy (TEM) analysis was used to obtain information on the matrix structure and morphology of NLC-SF. The samples were imaged using a XR41M mid-mount AMT camera on a Hitachi H-7650 TEM operating at 100 keV. A total of 10 µL of sample was placed on parafilm for 5 min to stabilize. After this time a 100 mesh formvar and carbon-coated copper grid were placed on the sample for 5 min and subsequently stained with a 2% solution of uranyl acetate for 3 min. The excess solution was removed with a piece of Whatman 1 filter paper, and the grids were left to air dry.

### 2.5. Lyophilization of NLC-NPOs

Samples of NLC-NPO were freeze-dried under vacuum using a lyophilizer Edwards Micromodul (BOC Ltd., Crawley, UK). A cooling rate of 1 °C/min was used to pre-cool the sample from room temperature to −50 °C, and the sample was maintained at −50 °C for 24 h. Since the primary purpose of drying the NLCs was to obtain a powder for further crystallinity evaluations, no matrix formers were added to the solutions prior to freeze-drying.

### 2.6. Evaluation of the Lipid Matrix Crystalline State by Differential Scanning Calorimetry

Differential scanning calorimetry (DSC) analysis was performed to analyze the crystalline state of NLC-NPOs with two SL:LL ratios. The thermograms were recorded using a DSC Q200 F3 (TA Instruments Inc., New Castle, DE, USA). A nitrogen purge provided an inert gas atmosphere within the D cell at a flow rate of 50 mL/min. A constant cooling rate of 10 °C/min was applied. Approximately 5–6 mg of dried NLC-NPO sample was hermetically sealed into standard aluminum pans. An empty pan was used as a reference. The samples were equilibrated at 0 °C and then submitted to a heating cycle from 0 to 350 °C with 10 °C/min heating rate. The melting points (Mp, °C) and enthalpies (ΔH, J g^−1^) were evaluated using the TA Universal Analysis 2000 (v4.5.0.5) software. The determination of the crystallinity index (*CI,* %) was calculated as follows:

(1) $$CI,\;\left(\%\right)=\frac{\Delta H_{NLC\text{-}NPO}}{\Delta H_{MA}}\times 100$$

where Δ*H_NLC_*_-_*_NPO_* and Δ*H_MA_* are the enthalpies of fusion of the NLCs and myristic acid, respectively.

### 2.7. Cytotoxicity

The cytotoxicity tests were performed for two dermal cell lines: Immortalized human keratinocytes (HaCaT) and Human Dermal Fibroblasts, neonatal (HDFn). HaCaT and HDFn were cultured in 175 cm^2^ flasks using Dulbecco’s modified Eagle’s medium (DMEM) supplemented with 10% fetal bovine serum (FBS) and 0.1% Pen Strep (10,000 U/mL penicillin, 10 µg/mL streptomycin). Cells were maintained at 37 °C in a 95% air/5% CO_2_ atmosphere and were detached with a trypsin solution (Trypsin/EDTA Solution, Gibco™). The cell lines were harvested at 80% confluence and were seeded in each well of 96-well plates at a density of 2 × 10^4^ cells/well. Cells were grown for 24 h at 37 °C in a 95% air/5% CO_2_ atmosphere to obtain subconfluence. Then, cells were washed with PBS solution and subsequently put in contact with 200 μL of each sample. NLCs samples were diluted with DMEM medium in 1/10, 1/20, 1/30, 1/40, 1/50, and 1/100 *w*/*w* ratios. For the positive control, cells were kept in contact only with the culture medium. The cytotoxicity of the developed formulations (NLCs) was evaluated, 3h after exposure, by the (MTT) reduction assay. MTT is a tetrazolium dye that is converted into formazan by metabolically active cells [46] Briefly, 10 µL of the 12 mM MTT was added to each well with the sample and incubated in a humidified 5% CO_2_-95% air atmosphere for 3 h. After this incubation period, the samples were removed from the wells, washed with PBS to remove unreacted MTT, and the formed formazan crystals dissolved in 100% dimethyl sulfoxide (DMSO) for 10 min. The absorbance was measured at 540 nm in a multi-well plate reader (SpectraMax 340PC Microplate Reader, Molecular Devices, LLC., San Jose, CA, USA). The cell viability (%) was calculated using the following equation:

(2) $$Cell\;viability,\;\%=\frac{Absorbance\;of\;treated\;cells}{Absorbance\;of\;negative\;control}\times 100$$

where the negative control was the cells incubated with DMEM medium alone.

### 2.8. Proliferation Test

The proliferation test was also performed with HaCaT and HDFn cell lines. In each well of a 96-well plate, 2 × 10^4^ cells/well were seeded and grown for 24 h to obtain subconfluence. Subsequently, the cells were washed in fresh medium and then put in contact with 200 μL of each NLC sample. NLCs were diluted 1/100 in DMEM medium. As a negative control, cells in contact with DMEM were used. The MTT assay was performed after 48 h, as described in Section 2.6.

### 2.9. Antimicrobial Activity Assays

Antimicrobial properties of NLCs were assessed against *Staphylococcus aureus* reference strain COL (Methicillin-resistant *S. aureus* isolated from infection, common lab strain). The minimum inhibitory concentration of NLCs was determined using the microdilution method [47]. Briefly, 100 μL of each NLC-NPO was added to well 1 with 100 μL Tryptic Soy Broth (TSB, Difco) bacterial growth medium, then serially diluted until well 10 in a 96-well plate. Well 11 served as a control with no NLC-NPO added and well 12 as a sterility control. Each well (1–11) was then inoculated with *S. aureus* (5 × 10^3^ cells), and the plates were incubated at 37 °C, with constant shaking, for 16 h. OD_600nm_ was measured every 30 min in a plate reader BioTek Synergy Neo2 (BioTek U.S., Winooski, VT, USA). Each experiment was repeated in triplicate.

### 2.10. Confocal Laser Scanning Microscopy (CLSM)

For CLSM imaging, cells were prepared on coverslips inside 6-well plates at a density of 1 × 10^5^ cells per well and incubated 24 h at 37 °C and 5% CO_2_. Cells were then incubated with DiO-labeled NLCs for 3 h. After incubation, cells were washed twice with PBS and fixed with 10% formalin for 10 min at room temperature. After rinsing twice with PBS, coverslips were stained with 5 µg/mL of WGA-Alexa 594 for 10 min at 37 °C. Then, cells were rinsed twice with PBS and nuclei stained with 300 nM DAPI for 5 min at room temperature. Then, coverslips were inverted over microscope slides using Vectashield as a mounting medium for fluorescence photobleaching prevention. CLSM images were acquired on a Leica SP5 CLSM (Leica Microsystems, Wetzlar, Germany) and processed using a Leica Application Suite—LAS AFv4.3 software.

### 2.11. Fluorescence Microscopy of S. aureus with NLC-NPOs

Interaction of NLCs with bacteria was observed by wide-field fluorescence microscopy using a Zeiss Axio Observer microscope with a Plan-Apochromat 100×/1.4 oil Ph3 objective. Images were acquired with a Retiga R1 CCD camera (QImaging) using Metamorph 7.5 software (Molecular Devices, San Jose, CA, USA). Bacteria were visualized using membrane stain Nile Red (5 µg mL^−1^, Invitrogen) and DNA stain Hoechst 33342 (1 µg mL^−1^, Invitrogen). NLC particles were labelled with DiO. Bacteria were grown to mid-exponential phase (OD_600nm_ 0.6–0.8), and labelled NLCs were added to 1 mL of culture and incubated for 30 min–3 h. Cells were then collected by centrifugation, washed with PBS and membranes and DNA labelled for 5 min at 37 °C. Cells were washed and placed on a thin layer of agarose on a microscopy slide for visualization.

### 2.12. Statistical Analysis

All data are reported as mean ± SD (standard deviation). NLC-NPO production and characterization as well as biocompatibility studies were performed at least in triplicate (*n* = 3). Results were submitted to variance analysis (ANOVA) with a level of significance of 95% using statistical discovery software JMP.

## 3. Results

### 3.1. Optimized NLC-NPOs with Stable Physicochemical Characteristics

An extensive physicochemical optimization was performed in previous work [11,12] for sunflower, olive, coconut, and sweet almond oil to encapsulate different vitamins. As a result, it was shown that the systems with the SL:LL = 60:40 and SL:LL = 40:60 lipid ratios had the best characteristics regarding the stability of the lipid carriers. Therefore, it was necessary to perform additional studies of the new NLC-NPO systems engineered in this work regarding physicochemical stability for these two lipid ratios. The objective was to establish stable, homogeneous NLC formulations with optimal surfactant concentration to avoid unwanted phenomena (e.g., excess of surfactants, particle aggregation) interfering in biocompatibility and antimicrobial studies. As a case of study for the surfactant concentration, based on previous results, the NLC-SF system was chosen.

#### 3.1.1. Surfactant Concentration Influence Stability of the System

**Figure 1:**
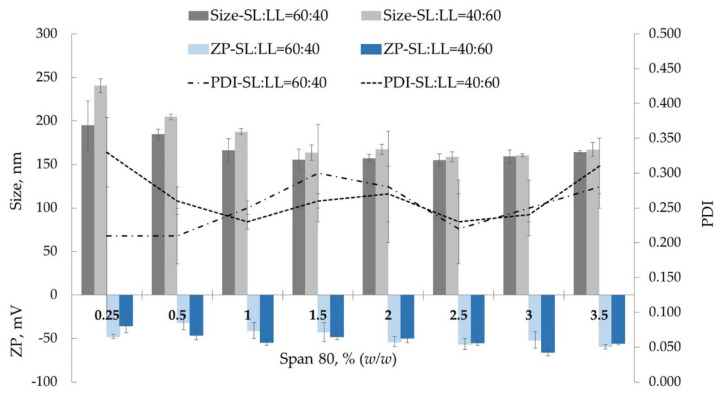
Influence of Span 80 concentration on particle size on NLC-SF.

The average size, PDI, zeta potential, and pH values of NLC formulations tested are reported in Figure 1. From the previous studies, it was demonstrated that the type of surfactant had a strong influence on the particle size, size distribution, and surface charge of the NLC-NPO. Previously, we showed that NLC-SF with 1.5% of Span 80 (HLB = 4.3) and NLC-SF with SL:LL = 80:20 led to good electrostatic and steric stabilization of the system [12]. The particle size was also significantly affected by the surfactant concentration used to stabilize the nanostructure [11,12,48,49]. Because of the crucial impact of surfactants on lipid carriers’ physicochemical characteristics, it was necessary to optimize surfactant concentration for SL:LL ratios 60:40 and 40:60. The particle size (Figure 1) was statistically significant and ranged between 155.1 ± 7.26 nm for SL:LL = 60:40 and 240.6 ± 7.85 nm for SL:LL = 40:60, with *p*-values of 0.048 and 0.016, respectively, at a significance level α = 0.05.

Irrespective of the SL:LL ratio, a decrease in the size from 194 ± 28.22 nm (SL:LL = 60:40) and 240.6 ± 7.85 nm (SL:LL = 40:60) for surfactant concentration 0.25% (*w*/*w*) to up to 155.1 ± 12.22 nm (SL:LL = 60:40) and 163.6 ± 8.85 nm (SL:LL = 40:60) for 1.5% (*w*/*w*) was observed. An additional increase in surfactant concentration did not significantly change the particle size. The standard deviation for these systems showed a higher value than the system with 2% (*w*/*w*) of Span 80 (157.1 ± 4.31 nm for SL:LL = 60:40 and 167.5 ± 5.7 nm for SL:LL = 40:60). There is an optimum level of surfactant concentration, which reduces the surface tension between the lipid and aqueous phases, leading to particles with smaller sizes and, consequently, to an increase in the surface area [49]. However, when this optimum level of surfactant concentration is overcome, saturation could be attributed to the accumulation of excess surfactant molecules on the NLC surface, preventing further reductions in particle size [11,12,49].

The particle size distribution characterized by PDI measures the degree of non-uniformity of the size distribution of particles. A PDI of 0.3 and below for lipid-based carriers indicates a homogenous population [49,50]. NLC-SF with SL:LL = 40:60, and 0.25% and 3.5% of surfactant PDI, was slightly higher than 0.3 (0.33 and 0.31, respectively), indicating a fairly narrow size distribution of the particles.

Zeta potential (ZP) is an important variable that enables prediction of the physical stability of dispersions, and higher values of ZP (≥|30|) tend to stabilize the NLC dispersion and avoid aggregation phenomena due to electrostatic repulsions between particles [11,12,48,49]. ZPs of all systems with both SL:LL ratios were between −36 mV ± 7.89 (SL:LL = 40:60; Span 80 = 0.25% *w*/*w*) and −66 mV ± 3.89 (SL:LL = 40:60; Span 80 = 3% *w*/*w*) (Figure 1). A slight increase in ZP from 0.25% (−48 mV ± 2.20 for SL:LL = 60:40 and −36 mV ± 7.89for SL:LL = 40:60) to 2% of Span 80 (−54 mV ± 6.06 for SL:LL = 60:40 and −50 mV ± 5.21for SL:LL = 40:60), despite the further increase in Span 80 concentration, did not show a significant effect.

**Figure 2:**
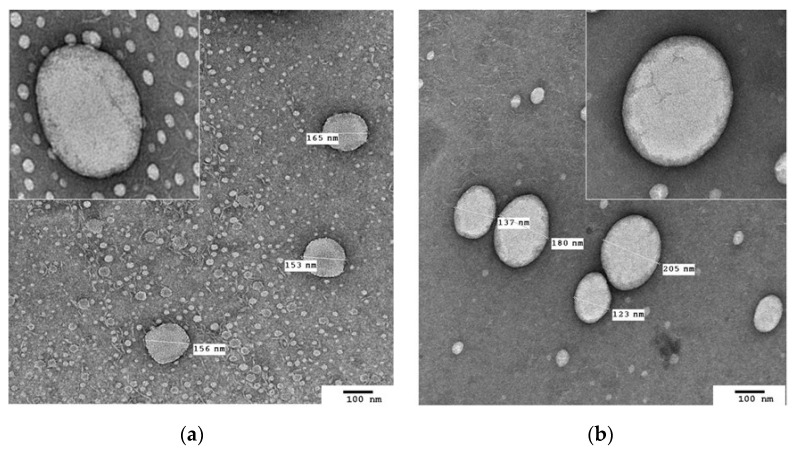
TEM micrographs of NLC-SF formulated with 2% of Span 80 as surfactants, sunflower oil, and myristic acid (C14:0) in (**a**) NLCs formulated with SL:LL = 60:40 (wt%), (**b**) NLCs formulated with SL:LL = 40:60 (wt%).

The morphology of NLCs prepared using SF oil with myristic acid (C14:0), 2% of Span 80 as a surfactant, and two SL:LL ratios 60:40 and 40:60 (wt%) were evaluated by TEM analysis, and micrographs are presented in (Figure 2).

The NLC particles displayed a spherical shape and particle sizes in the nano range. We can observe a typical solid–liquid lipid combination, where a fraction of the liquid lipid attached to the surface of the solid matrix as a liquid film or a liquid droplet forming NLCs, which facilitates incorporation of bioactive compounds and influences particle size [43,44]. As for NLC-SF (SL:LL = 60:40), a smaller size was observed compared to NLC-SF (SL:LL = 40:60), with mean diameters of 157.6 ± 4.31 nm and 160.5 ± 13.6 nm, respectively (Figure 1). Micrographs of the two systems (Figure 2a,b) showed this higher particle diameter observed for SL:LL = 40:60 system. Neither NLC-SF60:40 or NLC-SF40:60 systems presented cubic forms or sticks in their micrographs, corresponding to the β polymorphic transition, as expected for NLC [51]. However, slightly larger particles (approximately 6%) and no significant difference between PDI and ZP were observed for NLC-SF with 2% (*w*/*w*) of surfactant for two different SL:LL ratios. For further studies with NLC formulations with the new seven oils, this concentration of surfactant was used.

#### 3.1.2. Stable NLC-NPOs Are Obtained with All Selected Oils

The effects of the seven fixed oils and eucalyptus oil in the preparation of NLCs using Span 80 (2%, *w*/*w*) as a surfactant and myristic acid (C14:0) as a solid lipid on the particle size and physical stability for two SL:LL ratios were evaluated. The bibliographic overview of the composition of the oils is presented in Table 2.

**Figure 3:**
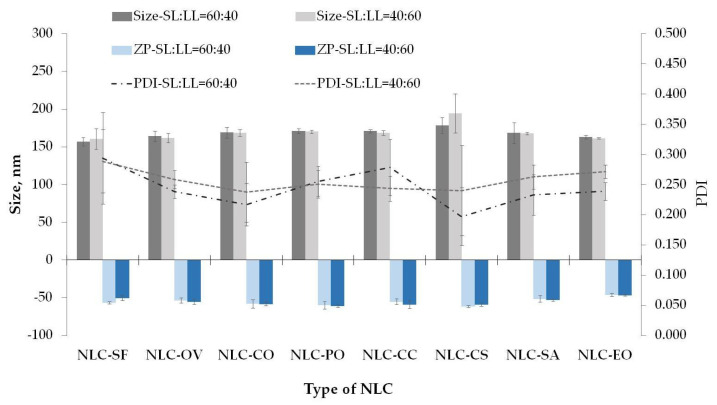
Physicochemical stability for NLC-NPOs after 24 h; Span 80, 2%, *w*/*w*.

The mean particle size, the PDI, and zeta potential of the lipid nanocarriers are illustrated in Figure 3. The particles size was statistically significant and ranged between 156.8 ± 5.52 NLC-SF (SL:LL = 60:40) and 194.5 ± 25.65 nm for NLCs-CS (SL:LL = 40:60). No significant difference (≤2.3%) was found for the particle size by changing the SL:LL ratio, which could be attributed to experimental error. An exception was found for the NLC-CS, where an increase of approximately 8% was observed for the system with SL:LL = 40:60. Gonzalez-Mira et al. [35] obtained a flurbiprofen (FB) loaded NLC formulation composed of 0.05 (wt%) FB, 1.6 (wt%) Tween 80, and 50:50 ratios of stearic acid to castor oil, with an average diameter of 288 nm, PI of 0.245, and ZP of −29 mV. Jawahar et al. [36] obtained olanzapine-loaded NLC-CS systems with an average size of 158.5 nm and PDI of 0.115, indicating narrow particle size distribution. According to published work [11,12], the average size of the fixed oil NLCs decreased in general with the increase in the liquid oil percentage, which was not observed in this study. The difference observed for castor oil may be ascribed to the high viscosity of the castor oil due to the presence of ricinoleic acid (Table 2), which interferes in experimental reproducibility. A high standard deviation was observed for both SL:LL ratios (178.8 ± 10.44 for SL:LL = 60:40; 194.5 ± 25.65 nm for SL:LL = 40:60). The particle size change by the type of oil used in NLC formulation is shown in Figure 3. However, the variation in particle size for all NLC-NPOs, except for NLC-CS, was ≤8.4% and ≤5.7% with SL:LL = 60:40 and SL:LL = 40:60, respectively. For NLC-CS this difference was 12% and 17% for SL:LL = 60:40 and SL:LL = 40:60, respectively, compared with the system with the lower particle diameter (NLC-SF).

The obtained polydispersity indices (from 0.197 to 0.294 for all formulations) revealed the lipid nanoparticles’ relatively uniform size distribution (Figure 3). The acquired ZP values ranged between −46.3 mV (NLC-EO; SL:LL = 60:40) and −61.3 mV (NLC-CS; SL:LL = 60:40) and indicated high physical stability [11,12,49]. Physicochemical characterization was repeated after 7 days and 1 month (data not shown). During one month, the particle average size of all systems changed less than 5%, with maximum variation for NLC-CC (SL:LL = 40:60; 4.1%). A maximum change for PDI (5.3%) was observed for NLC-OV (SL:LL = 40:60), keeping the PDI value below 0.3. The higher fluctuations were observed for zeta potential values for all systems. A significant increase of 23% after 1 month was observed for NLC-CC (SL:LL = 40:60), from −59.1 mV to −45.7 mV. PDI, in this case, increased 4.1% but still was lower than 0.3. The particle size change was 4.2%. After 7 days, the ZP variation for this system was 3.8%.

To provide stable and homogeneous NLC-NPOs for further biocompatibility and antimicrobial studies, samples not older than 7 days were used.

### 3.2. Crystallinity Studies

DSC is a consistent method used to determine possible interactions between the drug and lipid matrix and interactions between different liquid and solid lipids [51,52]. Verification of the solid state in lipid carriers showed that amorphous structures better supported drug loading and release [53]. DSC analysis was performed to evaluate the changes in the crystalline state of free NLC-NPOs using the selected oils. Based on previous optimization [11,12] we selected myristic acid (C14:0) as a solid lipid. Bibliographic information on the fatty acid composition of selected oils is presented in Table 2.

**Figure 4:**
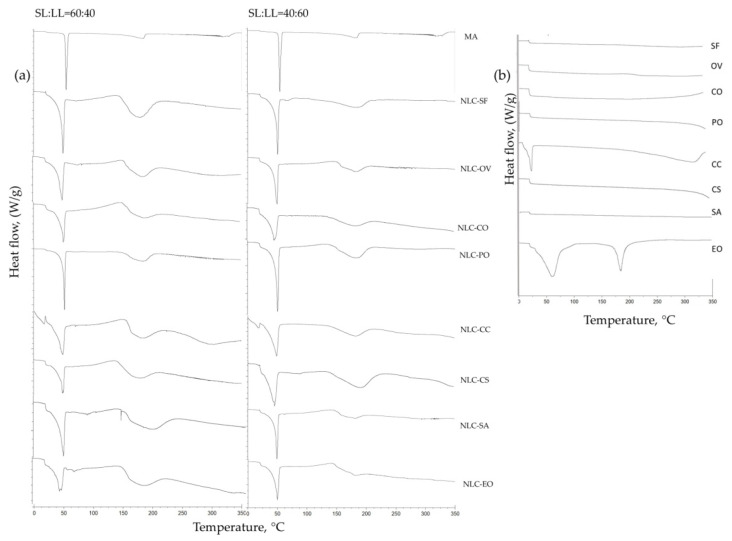
DSC thermogram: (**a**) for MA and NLC-NPO systems with two SL:LL ratios; (**b**) for pure oils.

Incorporating different oils with myristic acid as a solid lipid led to modifications in the physical state or crystallinity of myristic acid (C14:0). These lipid modifications in the nanocarrier matrix correspond to determining losses of energy measured as a function of temperature. DSC thermograms and determined parameters (melting temperatures (Tm, °C), melting enthalpies (ΔH, j/g), and the crystallinity index (CI, %)) of optimized formulations of all NLC-NPOs with the two SL:LL ratios are presented in Figure 4a and Table 4. DSC thermograms for pure oils are given in Figure 4b.

The melting point depression compared with the pure solid lipid (MA, 55.7 °C) was observed for all systems (Table 4). A decrease in the onset and end set temperatures (Ton, Tes) comparing with MA (Ton = 53.2 °C and Tes = 62.9 °C) was also observed for all systems (Figure 3). The width of each melting peak refers to the temperature span, mainly from the onset point to the ending point of the melting process, which increased with oil percentage in the lipid blends that was attributed to the less structured matrix. This was related to a disorder in the crystal order, corresponding to the development of more imperfections in the lipid matrix [52].

2A decrease in Tm between 4 °C (NLC-PO, SL:LL = 60:40, Tm = 51.7 °C) and 6.6 °C (NLC-OV SL:LL = 60:40, Tm = 51.7 °C) was observed for NLC-NPO made with SF, OV, CO, PO, and SA oils (Table 4), with similar saturated/unsaturated lipid ratios, where the saturated fatty acid percentage was between 10 and 16% (Table 2 and Table 3). For these systems, obtained melting enthalpies and CI (%) showed a decrease with the increase in the oil phase in the formulation. At the same time, different lipid ratios influenced insignificantly the Tm (≤1.5 °C) and did not show any tendency regarding oil phase increase.

For the systems where the difference of saturated and unsaturated lipids in oils was higher, such as NLC-CC (approximately 95% saturated FA) and NLC-CS (approximately 98% unsaturated FA) (Table 2 and Table 3), the polymorphic transition was more distinguished, and endothermic peaks at lower temperatures were observed (Table 4). For example, for NLC-CC, an increase in the crystalline degree of 19% was observed for SL:LL = 40:60. This may be because coconut oil is made of approximately 50% lauric acid (C12:0) and approximately 20% myristic acid (C14:0) (Table 4), which will increase the contribution of unsaturated FA for NLC-CC with SL:LL = 40:60 ratio. On the other hand, castor oil had an excess of ricinoleic acid (around 90%), unsaturated hydroxy fatty acids (C18:1), and demonstrated noticeable decrease in Tm (46.1 °C) for SL:LL = 40:60, which indicates a more amorphous structure of the lipid carrier.

The endothermic peaks of the lipid matrix from the NLC-EO presented the most significant decrease in Tm (appx 11 °C) of all selected oils compared with MA. The eucalyptus oil as an essential oil contained a meagre amount (2%) of fatty acids in its composition [40]. Although, besides that, lipid content was almost equally divided between saturated and unsaturated FAs (Table 2 and Table 3). Furthermore, the DSC thermogram and parameters for this system (Figure 4, Table 4) suggest that the liquid lipid ratio did not interfere with the lipid matrix’s physical state (crystallinity).

The DSC results showed differences in the degree of crystallinity obtained with the free lipid nanocarriers prepared with different NPO oil, which varied between 25% (NLC-CC, SL:LL = 60:40) and 55% (NLC-PO, SL:LL = 60:40). NLC-NPO formulations with oils with a comparable ratio between saturated and unsaturated lipids (SF, OV, CO, PO, and SA) showed a similar crystallinity index. There was no significant change in DSC parameters by increasing the lipid liquid phase. The coconut and castor oil systems had the highest effect on decreasing the crystalline structure, where the increase in oil concentration also had a higher impact (CI increase 19% for NLC-CC and decrease 9% for NLC-CS).

The evidence of broad peaks in thermograms of all NLC-NPOs, shown in Figure 4, was consistent with the presence of different crystalline structures of polymorphic states with varying melting points and a decrease in the degree of crystallinity. The chemical nature of the liquid lipids influenced the melting behavior of the solid lipids in the NLC [51,52]. These lower percentages of crystallinity and the decrease in the energy for lipid modification compared with the bulk crystalline solid lipid indicate that the lipid composition of natural oils may have had an impact on crystalline structure, causing disturbance to the crystal lattice.

All obtained NLC-NPO formulations were solid at body temperature, and the melting temperature was higher than 40 °C, which is a requirement for topical administration [49].

### 3.3. Biocompatibility Studies

#### 3.3.1. Cytotoxicity

**Figure 5:**
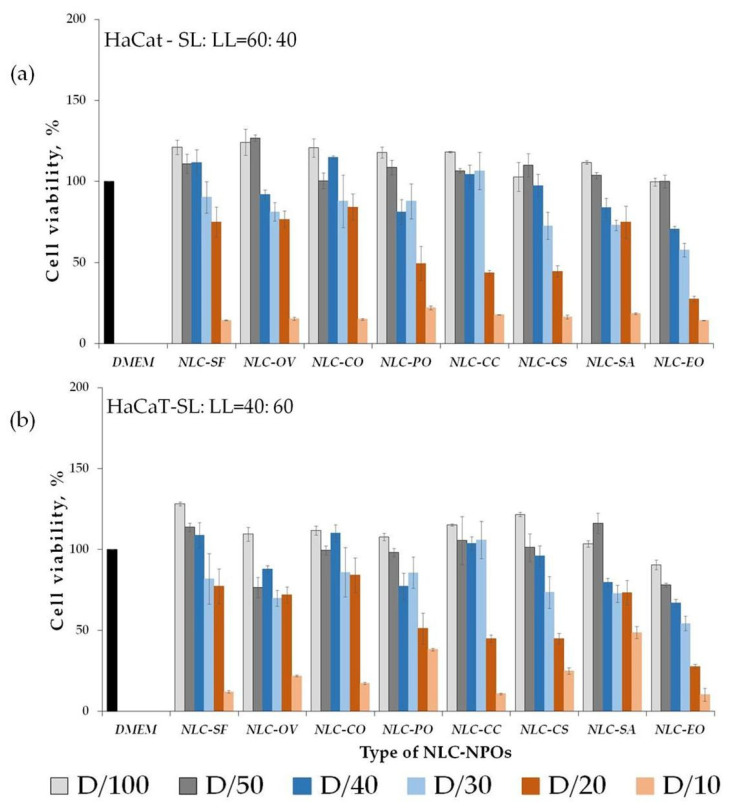
Cytocompatibility (viability %) of keratinocytes (HaCaT): (**a**) 60:40; (**b**) 40:60 of fibroblasts with all the nanoparticle suspensions; dilution (D) 1/10, 1/20, 1/30, 1/40, 1/50, and 1/100; Medium (DMEM) was used as comparison (positive control) (mean value ± SD; *n* = 3).

**Figure 6:**
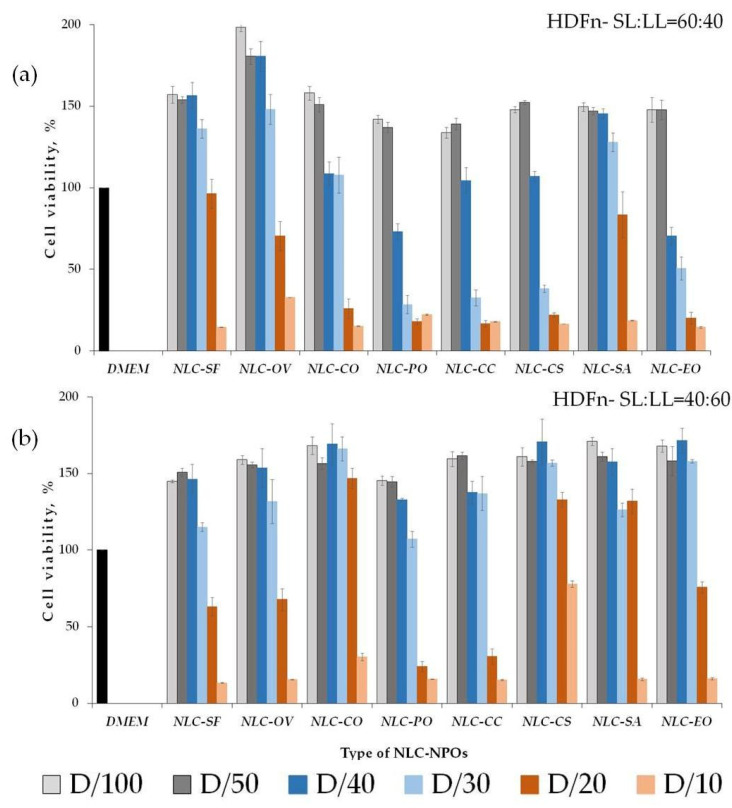
Cytocompatibility (viability %) of fibroblasts (HDFn): (**a**) 60:40; (**b**) 40:60 with all the nanoparticle suspensions; dilution (D) 1/10, 1/20, 1/30, 1/40, 1/50, and 1/100. Notes: Medium (DMEM) was used as comparison (positive control) (mean value ± SD; *n* = 3).

NLCs have provided controlled release profiles through different epithelia. They may be composed of physiological and biodegradable lipids, generally recognized as safe (GRAS) [1,2,3], thus improving biocompatibility. Besides that, bioavailability studies have shown some cytotoxicity expressed by unloaded control nanoparticles [54,55]. Determination of cell viability was used to test the biocompatibility of lipid nanoparticles. The keratinocytes play a vital role in immune skin response, while fibroblasts, as a major cellular part of the dermis, are responsible for hydration and elasticity [56]. Thus, it was important to evaluate the cytotoxicity of both cell lines (HaCaT) and fibroblasts (HDFn) in an in vitro experimental model. In an attempt to understand the role of the composition of NLC in terms of cytotoxicity, the effect of NLC-NPO formulations on cell viability after 24 h of incubation was studied in vitro on HaCaT and HDFn cell lines using the MTT assay for two SL:LL ratios. These two cell lineages have been used in well-defined experimental models in several pharmacology studies [56]. The obtained results are illustrated in Figure 5 and Figure 6, respectively. Regarding ISO 10993-5:2009, a reduction in cell viability by more than 30% was considered a cytotoxic effect [57].

All formulations showed a concentration-dependent effect, with toxicity increasing proportionally to the NLC concentration (Figure 5 and Figure 6). Nontoxic concentration of NLC-NPO systems for both cell lines, expressed by the total lipid ratio, is presented in Table 5. As shown in Figure 5, an increase in the oil phase in NLC formulations on HaCaT cells did not affect the cell viability for dilutions D ≥ 1/30 for both SL:LL ratios.

Table 5 shows the same cytotoxicity effect (nontoxic total lipid concentration, mg/mL^−1^) for both SL:LL for all NLC-NPOs. Higher toxicity was observed for NLC-EO, where the absence of toxicity was observed below 0.625 mg/mL^−1^. A lower cytotoxic effect was observed for the NLC-SF, NLC-OV, NLC-CO, and NLC-SA (total lipids below 1.25 mg/mL^−1^).

Fibroblasts showed a more pronounced response with the change of SL:LL ratio than keratinocytes. An increase in liquid lipids decreased the cytotoxic effect of particles (Figure 6, Table 5). It was observed that, despite the same nontoxic total lipid concentration for NLC-SF, NLC—OV, NLC-SA (≤1.25 mg/mL^−1^; both SL:LL ratios), NLC-PO, NLC-CC (≤0.833 mg/mL^−1^; SL:LL = 40:60), and NLC-EO (≤0.625 mg/mL^−1^; SL:LL = 60:40), the percentage of viable cells was higher for HDFn compared to HaCaT (Table 5).

For the HDFn cell line, cytotoxic concentrations of five systems (NLC-CO, NLC-PO, NLC-CC, NLC-CS, and NLC-EO) depended on the SL:LL ratio. For these systems, an increase in the oil phase decreased the cytotoxic effect of lipid carriers (Table 5).

Doktorovova et al. [54,55] reported that most cell lines tolerate lipid particle doses up to 1 mg/mL. Very few reports of cells surviving doses higher than 1 mg/mL exist. All of these formulations have a negative surface charge and are composed of excipients with known safety profiles.

NLCs as heterogeneous systems may form aggregates in cell culture media or undergo changes to their structure (e.g., desorption of surfactants).

**Figure 7:**
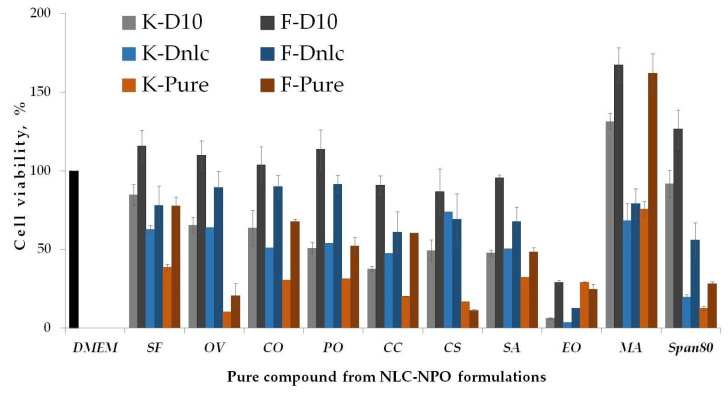
Cytotoxicity of compounds involved in NLC-NPO formulations on dermal cells; K—keratinocytes (HaCaT), F—fibroblasts (HDFn); Dnlc—concentration in final formulation; D10—dilution 10 of Dnlc; Pure compounds were taken in their original form. Medium (DMEM) was used as comparison (positive control) (mean value ± SD; *n* = 3).

Figure 7 shows cytotoxicity of all compounds involved in NLC formulations, pure, in quantities and ratios as in the final formulations (Dnlc) and in 10 dilutions of final formulations (D10). The lower value of cell viability of HaCaT cells compared to HDFn cells was also observed in this study. Myristic acid did not show cytotoxic effects on either cell line, indicating the influence of the oils in the composition upon both cell lines.

#### 3.3.2. Proliferation

Enhancements of the fibroblast’s proliferation influence collagen biosynthesis as well as dermal maturation, which is important for wound healing [58]. There is also a direct association between fibroblasts number and keratinocyte growth [59].

**Figure 8:**
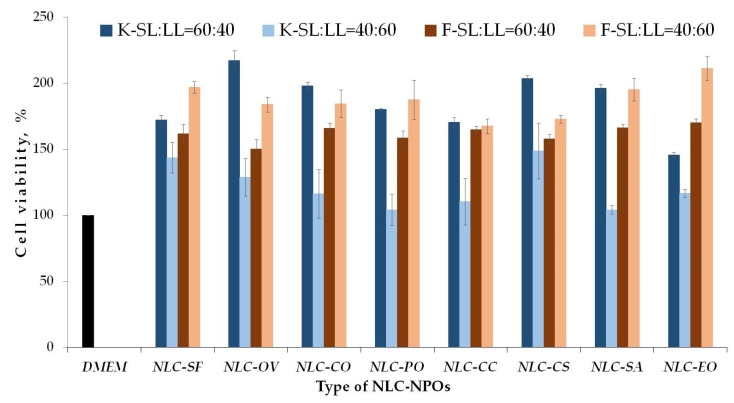
Proliferation (% viability) of HaCaT and HDFn in the presence of NLC-NPOs. K—keratinocytes (HaCaT), F–fibroblasts (HDFn); Dilution factor is 1/100. Medium (DMEM) was used as comparison (positive control) (mean value ± SD; *n* = 3).

Proliferation (% viability) results of the all the nanosystems developed for both cell lines (HaCaT and HDFn) are reported in Figure 8. Nanoparticle suspensions were diluted 1/100, and their impacts on HaCaT and HDFn cell viability were compared after 48 h. For most observed nanosystems the cell proliferation differed from those of the control (DMEM). The exception was observed for HaCat cells that showed lower cell viability in the presence of NLC-PO with SL:LL = 40:60 ratios than the other systems.

The higher percentage of oils seems to negatively influence HaCat cell proliferation and favor cell viability for HDFn cells for different NLC-NPO systems. The lower proliferation of HaCat cells was observed for NLC-PO, NLC-CC, NLC-SA, and NLC-EO. This can be related to concentration-dependent fatty acid cytotoxicity already observed in HaCaT [60]. Cell viability for HDFn cell lines increased with higher participation of the oil phase for all NLC-NPOs. Khezri et al. [30] showed that the essential oil loaded NLC system reduced tissue bacterial colonization, while they increased fibroblast infiltration and re-epithelization.

#### 3.3.3. Internalization Analysis by Fluorescence Microscopy

It was reported that the physicochemical characteristics and composition of nanoparticles influence their interaction with eukaryotic cells [61,62]. As the dermal absorption of lipid particles involves direct contact with the stratum corneum of the skin, it was essential to screen the cellular uptake of NLC-NPOs by keratinocytes. However, there are no comparative studies of empty lipid particles on cellular uptake for topical delivery based on different lipid compositions. In previous studies the cellular uptake was mainly investigated for drug-loaded lipid carriers [61,62,63,64]. We chose for our experiments five systems selected by their differing fatty acid composition (Table 1 and Table 3). The chosen NLC-NPOs were labelled with DiO and incubated with HaCaT cells. Their uptake was observed after 3 h by Confocal Scanning Laser Microscopy (CSLM). The incubation conditions, 3 h exposure time, and NLC-NPO dilution (1:50) were used, as we previously showed that all NLC-NPOs are nontoxic for HaCaT cells under these conditions (Figure 5).

**Figure 9:**
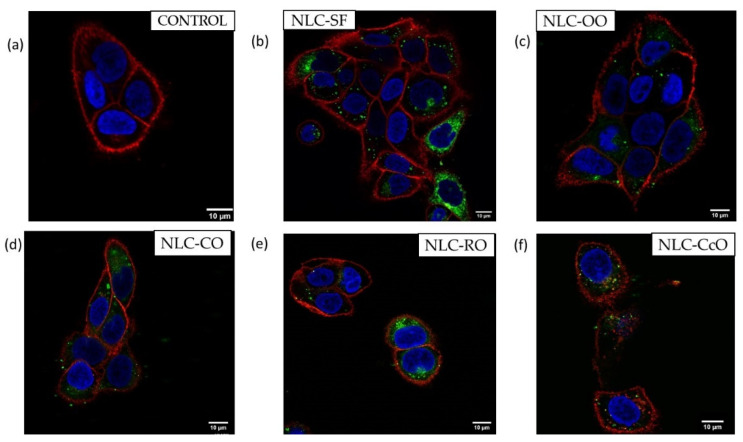
Cellular internalization of five different NLCs at dilution D = 50 after 3 h of incubation. (**a**) HaCaT cells; (**b**) NLC-SF; (**c**) NLC-OV, (**d**) NLC-CO, (**e**) NLC-CS and (**f**) NLC-CC.

In Figure 9 CLSM images show cell membranes labeled with WGA-Alexa 594 (red) and nuclei stained with DAPI (blue). HaCat cells cultured with non-labeled lipid nanoparticles showed no green fluorescence (Figure 9a). The fluorescence micrographs showed intracellular localization of all tested NLC-NPOs, which accumulated around the nuclei (Figure 9b–f). These results indicate the potential use of NLC systems for the delivery of encapsulated drugs to specific intracellular target areas without causing damage to the cells. The cellular nucleus is the main target site for many therapeutic drugs [65]. NLCs appear in a perinuclear location, which might be important for higher drug concentration around the nucleus. The intracellular distribution of particles within the HaCaT cells also indicates their location in specific compartments, which is important as many drug targets are localized to particular subcellular compartments [65]. The differences observed in cellular uptake of the five NLC-NPOs are likely related to their different lipid compositions (Table 2 and Table 3) as the size and charge of both types of lipid nanoparticles were found to be similar (Figure 2). An evaluation of the crystallinity parameters in NLC-NPOs by DSC (Figure 3, Table 4) showed variations indicating different concentration lattice defects in the lipid matrix, which could suggest differences in their ability to accommodate potential drugs [66]. The perinuclear uptake of lipid particles has already been observed in keratinocytes [63,64]. Using CLSM, we confirmed the cellular uptake of NLC-NPOs under conditions that were shown to be non-toxic to human cells.

### 3.4. The Type of Oil Used in NLC Formulations Influences the Antimicrobial Effect

#### 3.4.1. Antimicrobial Effect NLC-NPO on *S. aureus*

All eight NLC-NPOs for both SL:LL ratios and pure compounds from nanoparticle compositions were tested for antimicrobial activity against *S. aureus* strain COL. There is extensive literature concerning the antibacterial effects of various FFAs from a wide range of biological sources, including algae, animals, and plants, and various microorganisms [17,67]. It was reported that multiple strains of *S. aureus* are susceptible to the antibacterial effects of unsaturated FFAs. Polyunsaturated linolenic acid (C18:3) can reduce *S. aureus* numbers on human skin [68]. Furthermore, Lukowski et al. [69] showed that emulsions of FA-rich extracts from microalgae reduced MRSA attachment to pre-treated skin. Regarding oils, a complex mixture of different fats and also bioactive compounds (e.g., polyphenolic compounds), there are also many examples in the literature of their antimicrobial activities [70]. In 2007 Kisich et al. [71] reported the capability of keratinocytes in the skin to bind, internalize, and mobilize defenses against bacteria. However, natural plant oils are also the source of other bioactive compounds with antimicrobial effects, so our particular objective was to analyze the degree of FFA’s antimicrobial impact on *S. aureus*. As previously mentioned, all chosen oils had different but comparable FFA content (Table 2 and Table 3), and solid lipid from NLC-NPOs formulations was a fixed parameter in all formulations.

Bacterial growth was monitored by measuring OD_600nm_ in a 96-well plater reader for 16 h with constant shaking at 37 °C. NLC-NPOs were serially diluted from Wells 1–10 into bacterial growth media inoculated with 5 × 10^3^ cfus. Well 11 contained only bacteria and showed normal growth for this *S. aureus* strain under these conditions. Well 12 acted as a sterility control. It can be seen that bacterial growth was inhibited in the presence of increasing concentrations of NLC-NPO and that the 60:40 ratio showed a slightly higher growth inhibition than the 40:60 ratio.

The ability of all NLC-NPOs, at both SL:LL ratios, to inhibit bacterial growth expressed by oil and total lipid concentration (mg/mL) is shown in Table 6. The results indicate that all systems had antimicrobial activity, and this varied depending on the type of lipid carrier. For SL:LL = 60:40, increased antimicrobial activity was observed for NLC-SF, NLC-OV, and NLC-CC, while NLC-PO and NLC-EO showed higher antimicrobial activity with the SL:LL = 60:40 ratios. Antimicrobial activities for NLC-CS and NLC-SA were equal for both lipid proportions.

The NLC-SF and NLC-CO systems, which have a very similar saturated vs. unsaturated composition (Table 1 and Table 3), showed a significant difference in antimicrobial activity, with NLC-SF showing a stronger inhibition on bacterial growth than NLC-CO. It may indicate that other bioactive compounds from NPO (e.g., polyphenolic compounds) compositions could have a higher antimicrobial impact than the present FFAs. The antimicrobial effect of polyphenolic compounds and their increased bioavailability by incorporating lipid nanocarriers is well reported in the literature [72]. For future studies the full composition of NPOs will be considered for antimicrobial effects.

**Figure 10:**
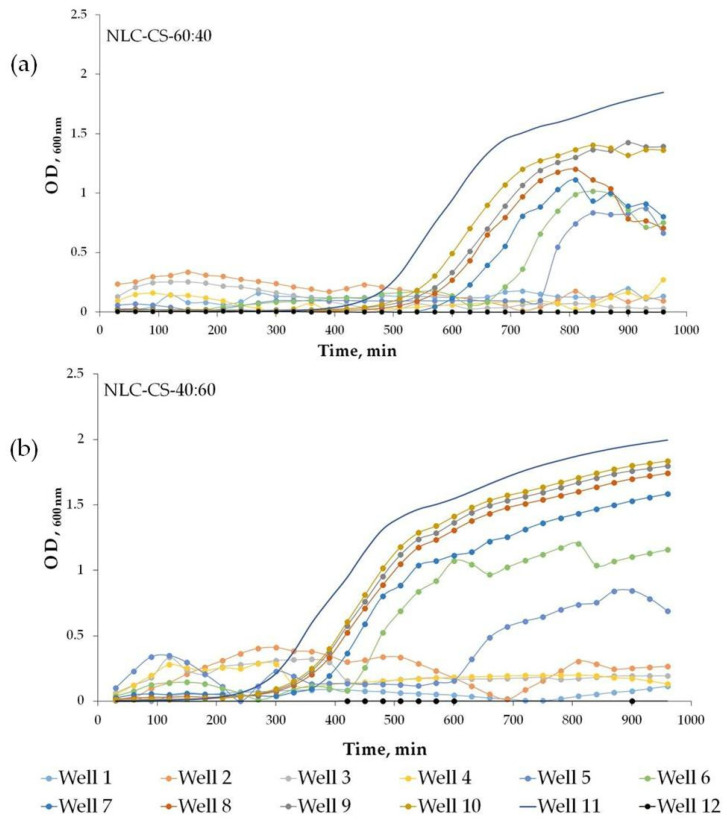
*S. aureus* growth in the presence of NLC-CS for (**a**) SL:LL = 60:40 and (**b**) SL:LL = 40:60 ratios; *S. aureus*: 5 × 10^3^ cells/well, incubation at 37 °C, with constant shaking for 16 h, OD_600 nm_ was measured every 30 min. Each experiment was repeated in triplicate.

Even for the systems where the same inhibitory concentration was observed for both SL:LL ratios, the bacterial growth curve kinetic had a different profile (Figure 10). For example, for NLC-CS, bacterial growth started after 12.5 h for SL:LL = 60:40, and after 10 h for SL:LL = 40:60 (Figure 10), suggesting the different oils affect bacterial growth to varying degrees.

Comparison of bacterial growth inhibition and cytotoxic effects on human cells at each NLC-NPO concentration is shown in Table 5 and Table 6. These data showed that in some systems, antimicrobial activity was only seen at concentrations shown to be cytotoxic to human cells. For example, it was observed for the NLC-CO, NLC-CS, NLC-SA, and NLC-EO systems for both SL:LL ratios, and for the NLC-PO/SL:LL = 60:40, and NLC-CC/SL:LL = 40:60 systems. Only the NLC-SF and NLC-OV systems showed antimicrobial activity at concentrations shown to be non-toxic for HaCaT cells.

#### 3.4.2. Interaction of NLC-SF with *S. aureus*

**Figure 11:**
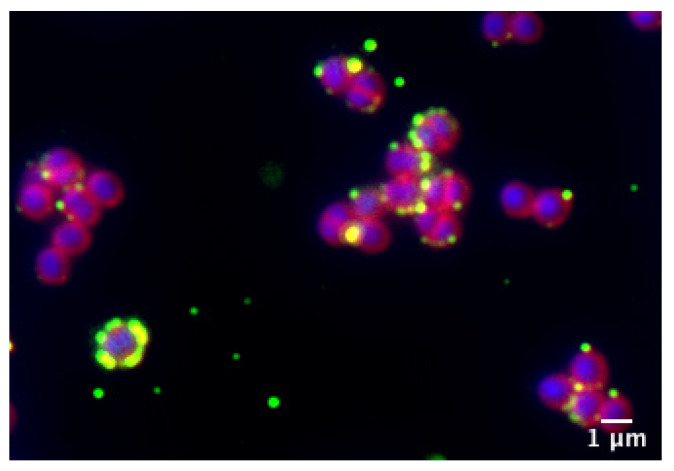
NLC-SF interacts with *S. aureus* cells in liquid culture; NLC-SF was stained with DiO (green), *S. aureus* membranes were labelled with Nile Red (red) and DNA was labelled with Hoechst (blue).

Fluorescently labeled (DiO) NLC-SF was added to exponentially growing *S. aureus* to observe NLC interaction with bacteria. At 1/50 dilution of NLC-SF (a concentration non-toxic to human cell lines) bacteria was mostly destroyed (images not shown). At 1/100 dilution of NLC-SF, labelled particles can be clearly seen close to/associated with the bacterial membrane (Figure 11).

**Figure 12:**
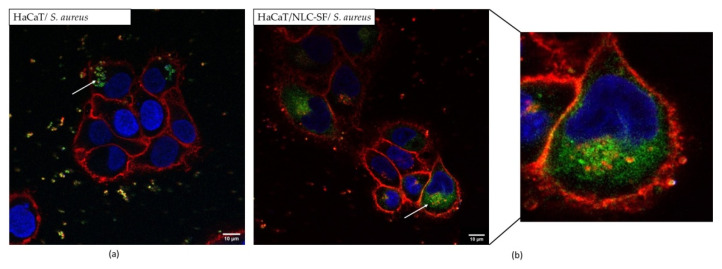
Cellular internalization (**a**) HaCat cells in the presence of SA-GFP; (**b**) HaCaT cells in the presence of *S. aureus* and NLC-SF; HaCaT were first in contact with *S. aureus* for 1 h and then 3 h of NLC exposure; NLC-SF dilution D = 50.

*S. aureus* cells remained normal after a 30 min incubation with NLC-SF prior to imaging (Figure 11); however, after 1–2 h cells began to die (as indicated by accumulation of red membrane staining (images not shown)). The mode of action of killing of NLC-NPOs is yet to be determined; however, this result indicates the particles interact with and possibly disrupt the bacterial membrane. The cellular internalization experiment was repeated in the presence of *S. aureus* infected HACaT cells (incubated for 1 h with *S. aureus* and washed to remove external bacteria, before addition of NLC-SF, as described in the materials and methods), in order to see if NLC-SF also interacted with bacteria in vivo. As can be seen in Figure 12a, *S. aureus* expressing GFP (SA-GFP) were internalized by HACaT cells after a 1 h incubation. In Figure 12b *S. aureus* cells are not expressing GFP; however, they are stained with WGA (which binds the bacterial cell wall) during the labeling of HACaTcells. It was observed that NLC-SF accumulated near the nucleus, as previously shown, and can also be seen accumulating around the bacterial cells inside of the HACaT cells. This observation suggests that the NLC-NPOs can exert their antimicrobial action inside human cells in the same way as in a liquid culture of bacteria. Future experiments will investigate optimal conditions for using NLCs to completely clear bacteria from within human cells.

## 4. Conclusions

Seven fixed oils and eucalyptus essential oil were selected and successfully applied in new formulations of multifunctional free NLCs for two SL:LL ratios with optimized surfactant concentration.

The choice of natural plant oil influenced the physicochemical stability by changing the diameter of NLC formulations (between 160 nm and 185 nm) and Z-potential (between −46 mV and −61 mV). There are differences in the degree of crystallinity obtained with the free NLCs prepared with different oils.

The FFAs composition of NPOs affects changes in the crystalline state of free NLC-NPOs for different SL:LL ratios.

Fibroblasts showed a more pronounced response with the change of SL:LL ratio than keratinocytes. Cytotoxicity of HaCaT cells was not influenced by different SL:LL ratios, while SL:LL = 40:60 ratio decreased toxicity of NLC-NPOs for HDFn cells. NLC-SF, NLC-CO, and NLC-SA were found to be less toxic for both cells lines. Using CLSM, we confirmed the cellular uptake of NLC-NPOs around the nucleus under conditions that were shown to be non-toxic to human cells.

The results indicate that all systems have antimicrobial activity, which varies depending on the type of lipid carrier. However, for only two systems, NLC-SF and NLC-OV, antimicrobial activity was observed at concentrations that were also non-toxic for both cell lines. The presence of fatty acids in NPOs did not show the major impact on the antimicrobial effect of NLC-NPOs. The cellular internalization of HaCaT in the presence of *S. aureus* and NLC-SF showed the perinuclear position of bacteria and particles, which make them a good nanocarrier for drug transport within the cell.

This research contributes to the development of safe, naturally based lipid nanocarriers with antimicrobial effects, which could enhance efficiency in drug conjugated systems and reduce their secondary effects and physicochemical instability.

For future studies, to understand better the impact of lipids on cellular interactions of NLC-NPOs and the transport mechanism, time- and particle concentration-dependent cellular uptake will be performed, and impacts of other bioactive compounds from NPOs structure will be analyzed.

## Figures and Tables

**Table 2:** Bibliographic information on the fatty acid composition of selected oils.

Oils		SF	OV	CO	PO	CC	CS	SA	EO ^2^
	FFAs,%								
C12:0		0.1	-	-	-	50–52	-	-	-
C14:0		0.1	-	-	0.6	19–21	-	0.07	-
C16:0		6.1–6.6	11–14	10–11	0.31–19	7.5–9.3	0.8–1.1	4.7–16	36
C18:0		3.3–5.3	1.6–2.6	1.8–2	0.1–4.9	2–7.8	0.7–1.0	0.3–3.0	3.36
C20:0		0.3	0.4	0.4	0.15–2.3	-	-	0.04–0.2	-
C22:0		0.6	0.1	0.1	0.13–4.5	-	-	-	-
C24:0		0.2	0.1	0.2	0.09–2.5	-	-	-	-
C16:1		0.1	0.1–1.5	0.1	0.6	-	-	-	-
C18:1		21–30	64–78	26–28	16–69	7–8.6	2.2–3.3/ 88–90 ^1^	50–86	27
C20:1		0.2	0.3	0.3	0.3–2.4	-	-	-	-
C18:2		58–66	6.2–16	56–60	14–60	1.8–9.2	4.1–4.7	-	19
C18:3		0.1–1.5	0.6–3.1	0.8–0.9	0.07	0.1	0.5–0.7	0.1–1.0	-
Ref.		[41,42,43]	[42,43]	[42]	[42,44]	[42,43]	[42,43]	[45]	[40]

^1^88–90% belong to Ricinoleic acid that is an 18-carbon hydroxylated fatty acid with one double bond; 2.2–3.3% belong to Oleic acid; ^2^ EO—essential oil with lower total lipid content compared to other analyzed fixed oils.

**Table 3:** Composition of NLC formulations with two SL:LL ratios.

Scheme	SL, wt%	LL, wt%	Surfactant, wt%
60:40	1.5	1	2
40:60	1	1.5	2

**Table 4:** Melting temperature (Tm, °C), enthalpy (ΔH, j/g), and crystallinity index (CI, %) of NLC-NPOs with different SL:LL ratios.

SL:LL	Tm, °C		ΔH, J/g		CI, %	
	60:40	40:60	60:40	40:60	60:40	40:60
NLC-SF	49.4	51.0	76.5	73.4	40	38
NLC-OV	49.1	49.7	83.6	82.2	44	43
NLC-CO	49.8	50.1	80.7	79.9	42	42
NLC-PO	51.7	50.4	110.3	101.0	58	53
NLC-CC	47.7	48.4	47.1	83.1	25	43
NLC-CS	48.4	46.0	69.7	51.6	36	27
NLC-SA	50.7	49.2	104.7	102.4	55	54
NLC-EO	44.3	45.5	67.9	70.5	36	37
Myristic acid	55.7		190.9		100	

**Table 5:** Nontoxic total lipid concentration (mg/mL^−1^) of NLC-NPOs for HaCaT and HFDn cell lines expressed by amount of total lipid (mg/mL^−1^).

Total Lipids, mg/mL^−1^	HaCaT	HFDn		
NLC-SF	1.25	1.25		
NLC-OV	1.25	1.25		
NLC-CO	1.25	0.833		1.25
NLC-PO	0.833	0.625		0.833
NLC-CC	0.833	0.625		0.833
NLC-CS	0.833	0.625		1.25
NLC-SA	1.25	1.25		
NLC-EO	0.625	0.625		1.25
SL:LL, wt%	60:40/40:60	60:40	60:40/40:60	40:60

**Table 6:** Antimicrobial effect of NLC-NPO on *S. aureus*.

Lipid Ratio	SL:LL = 60:40		SL:LL = 40:60	
NLC-NPOs	Last well in which growth was inhibited	Total lipids in well, mg/mL^−1^	Last well in which growth was inhibited	Total lipids in well, mg/mL^−1^
NLC-SF	6	0.39	5	0.78
NLC-OV	6	0.39	5	0.78
NLC-CO	3	3.13	2	6.25
NLC-PO	4	1.56	5	0.78
NLC-CC	5	0.78	4	1.56
NLC-CS	4	1.56	4	1.56
NLC-SA	4	1.56	4	1.56
NLC-EO	4	1.56	5	0.78

## Data Availability

The data presented in this study are available on request from the corresponding author.

## References

[1] Jaiswal P., Gidwani B., Vyas A. (2016). Nanostructured lipid carriers and their current application in targeted drug delivery. Artif. Cells Nanomed. Biotechnol..

[2] Chauhan I., Yasir M., Verma M., Pratap Singh A. (2020). Nanostructured Lipid Carriers: A Groundbreaking Approach for Transdermal Drug Delivery. Adv. Pharm. Bull..

[3] Beloqui A., Ángeles Solinís M., Rodríguez-Gascón A., Almeida A.J., Préat V. (2016). Nanostructured lipid carriers: Promising drug delivery systems for future clinics. Nanomedicine.

[4] Ferreira K.C.B., Valle A.B.C.d.S., Paes C.Q., Tavares G.D., Pittella F. (2021). Nanostructured Lipid Carriers for the Formulation of Topical Anti-Inflammatory Nanomedicines Based on Natural Substances. Pharmaceutics.

[5] Wang L., Hu C., Shao L. (2017). The Antimicrobial Activity of Nanoparticles: Present Situation and Prospects for the Future. Int. J. Nanomed..

[6] Gao W., Chen Y., Zhang Y., Zhang Q., Zhang L. (2018). Nanoparticle-Based Local Antimicrobial Drug Delivery. Adv. Drug Deliv. Rev..

[7] Pardeike J., Hommoss A., Müller R.H. (2009). Lipid nanoparticles (SLN, NLC) in cosmetic and pharmaceutical dermal products. Int. J. Pharm..

[8] Ghasemiyeh P., Mohammadi-Samani S. (2018). Solid lipid nanoparticles and nanostructured lipid carriers as novel drug delivery systems: Applications, advantages and disadvantages. Res. Pharm. Sci..

[9] Subramaniam B., Siddik Z.H., Nagoor N.H. (2020). Optimization of nanostructured lipid carriers: Understanding the types, designs, and parameters in the process of formulations. J. Nanoparticle Res..

[10] Yang Y., Corona A., Schubert B., Reeder R., Henson M.A. (2014). The effect of oil type on the aggregation stability of nanostructured lipid carriers. J. Colloid Interface Sci..

[11] Pinto F., de Barros D.P.C., Fonseca L.P. (2018). Design of multifunctional nanostructured lipid carriers enriched with α-tocopherol using vegetable oils. Ind. Crops Prod..

[12] Pinto F., de Barros D.P.C., Reis C., Fonseca L.P. (2019). Optimization of nanostructured lipid carriers loaded with retinoids by central composite design. J. Mol. Liq..

[13] D’Souza A., Shegokar R., Rai M., Zacchino S., Derita M. (2017). Potential of oils in development of nanostructured lipid carriers. Essential Oils and Nanotechnology for Treatment of Microbial Diseases.

[14] Lercker G., Rodriguez-Estrada M.T. (2000). Chromatographic analysis of unsaponifiable compounds of olive oils and fat-containing foods. J. Chromatogr. A.

[15] Bonaccorso A., Cimino C., Manno D.E., Tomasello B., Serra A., Musumeci T., Puglisi G., Pignatello R., Carbone C. (2021). Essential Oil-Loaded NLC for Potential Intranasal Administration. Pharmaceutics.

[16] Lin T.-K., Zhong L., Santiago J.L. (2018). Anti-Inflammatory and Skin Barrier Repair Effects of Topical Application of Some Plant Oils. Int. J. Mol. Sci..

[17] Desbois A.P., Smith V.J. (2010). Antibacterial free fatty acids: Activities, mechanisms of action and biotechnological potential. Appl. Microbiol. Biotechnol..

[18] Trommer H., Neubert R.H.H. (2006). Overcoming the Stratum Corneum: The Modulation of Skin Penetration, Skin. Pharmacol. Physiol..

[19] Dr Rimpler. http://cheerful.com.my/brands/dr-rimpler/.

[20] Sharma A., Madhunapantula S.V., Robertson G.P. (2012). Toxicological considerations when creating nanoparticle-based drugs and drug delivery systems. Expert Opin. Drug Metab. Toxicol..

[21] Pinilla C.M.B., Lopes N.A., Brandelli A. (2021). Lipid-Based Nanostructures for the Delivery of Natural Antimicrobials. Molecules.

[22] Parlet C.P., Brown M.M., Horswill A.R. (2019). Commensal Staphylococci Influence *Staphylococcus aureus* Skin Colonization and Disease. Trends Microbiol..

[23] Tong S.Y., Davis J.S., Eichenberger E., Holland T.L., Fowler V.G. (2015). *Staphylococcus aureus* infections: Epidemiology, pathophysiology, clinical manifestations, and management. Clin. Microbiol. Rev..

[24] Ki V., Rotstein C. (2008). Bacterial skin and soft tissue infections in adults: A review of their epidemiology, pathogenesis, diagnosis, treatment and site of care. Can. J. Infect. Dis. Med. Microbiol..

[25] Nichols R.L., Florman S. (2001). Clinical presentations of soft-tissue infections and surgical site infections. Clin. Infect. Dis..

[26] Van Bambeke F., Barcia-Macay M., Lemaire S., Tulkens P.M. (2006). Cellular pharmacodynamics and pharmacokinetics of antibiotics: Current views and perspectives. Curr. Opin. Drug Discov. Dev..

[27] Umerska A., Cassisa V., Matougui N., Joly-Guillou M.-L., Eveillard M., Saulnier P. (2016). Antibacterial action of lipid nanocapsules containing fatty acids or monoglycerides as co-surfactants. Eur. J. Pharm. Biopharm..

[28] World Health Organization—WHO Global Action Plan on Antimicrobial Resistance. https://www.who.int/publications/i/item/9789241509763.

[29] Da Silva L.C.N., da Silva M.V., Correia M.T.S. (2017). Editorial: New frontiers in the search of antimicrobials agents from natural products. Front. Microbiol..

[30] Khezri K., Farahpour M.R., Mounesi Rad S. (2019). Accelerated infected wound healing by topical application of encapsulated Rosemary essential oil into nanostructured lipid carriers. Artif. Cells Nanomed. Biotechnol..

[31] Saporito F., Sandri G., Bonferoni M.C., Rossi S., Boselli C., Icaro Cornaglia A., Mannucci B., Grisoli P., Vigani B., Ferrari F. (2017). Essential oil-loaded lipid nanoparticles for wound healing. Int. J. Nanomed..

[32] Zhang L., Pornpattananangkul D., Hu C.M.J., Huang C.M. (2010). Development of nanoparticles for antimicrobial drug delivery. Curr. Med. Chem..

[33] Brandelli A., Pinilla C.M.B., Lopes N.A., Rai M., Santos C.A. (2017). Nanoliposomes as a plataform for delivery of antimicrobials. Nanotechnology Applied to Pharmaceutical Technology.

[34] Huguet-Casquero A., Moreno-Sastre M., López-Méndez T.B., Gainza E., Pedraz J.L. (2020). Encapsulation of Oleuropein in Nanostructured Lipid Carriers: Biocompatibility and Antioxidant Efficacy in Lung Epithelial Cells. Pharmaceutics.

[35] Gonzalez-Mira E., Egea M.A., Garcia M.L., Souto E.B. (2010). Design and ocular tolerance of flurbiprofen loaded ultrasound-engineered NLC. Colloids Surf. B Biointerfaces.

[36] Jawahar N., Hingarh P.K., Arun R., Selvaraj J., Anbarasan A., Sathianarayanan S., Nagaraju G. (2018). Enhanced oral bioavailability of an antipsychotic drug through nanostructured lipid carriers. Int. J. Biol. Macromol..

[37] Yoon B.K., Jackman J.A., Valle-González E.R., Cho N.J. (2018). Antibacterial Free Fatty Acids and Monoglycerides: Biological Activities, Experimental Testing, and Therapeutic Applications. Int. J. Mol. Sci..

[38] Zuzarte M., Salgueiro L., de Sousa D. (2015). Essential Oils Chemistry. Bioactive Essential Oils and Cancer.

[39] Eromosele I.C., Eromosele C.O., Akintoye A.O., Komolafe T.O. (1994). Characterization of oils and chemical analyses of the seeds of wild plants. Plant Foods Hum. Nutr..

[40] Rekkab S., Zarrok H., Salghi R., Zarrouk A., Bazzi L., Hammouti B., Kabouche Z., Touzani R., Zougagh M. (2012). Green Corrosion Inhibitor from Essential Oil of *Eucalyptus globulus* (Myrtaceae) for C 38 Steel in Sulfuric Acid Solution. J. Mater. Environ. Sci..

[41] Douvartzides S.L., Charisiou N.D., Papageridis K.N., Goula M.A. (2019). Green Diesel: Biomass Feedstocks, Production Technologies, Catalytic Research, Fuel Properties and Performance in Compression Ignition Internal Combustion Engines. Energies.

[42] Cecilia J.A., Ballesteros Plata D., Alves Saboya R.M., Tavares de Luna F.M., Cavalcante C.L., Rodríguez-Castellón E. (2020). An Overview of the Biolubricant Production Process: Challenges and Future Perspectives. Processes.

[43] Mawatari T., Fukuda R., Mori H., Mia S., Ohno N. (2013). High Pressure Rheology of Environmentally Friendly Vegetable Oils. Tribol. Lett..

[44] Akhtar S., Khalid N., Ahmed I., Shahzad A., Suleria H.A. (2014). Physicochemical characteristics, functional properties, and nutritional benefits of peanut oil: A review. Crit. Rev. Food Sci. Nutr..

[45] Fernandes G.D., Gómez-Coca R.B., Pérez-Camino M.C., Moreda W., Barrera-Arellano D. (2017). Chemical Characterization of Major and Minor Compounds of Nut Oils: Almond, Hazelnut, and Pecan Nut. J. Chem..

[46] Mosmann T. (1983). Rapid colorimetric assay for cellular growth and survival: Application to proliferation and cytotoxicity assays. J. Immunol. Methods.

[47] Andrews J.M. (2001). Determination of minimum inhibitory concentrations. J. Antimicrob. Chemother..

[48] Araujo V.H.S., Bento da Silva P., Oliveira Szlachetka I., William da Silva S., Fonseca-Santos B., Chorilli M., Ganassin R., de Oliveira G.R.T., da Rocha M.C.O., Fernandes R.P. (2020). The influence of NLC composition on curcumin loading under a physicochemical perspective and in vitro evaluation. Colloids Surf. A Physicochem. Eng. Asp..

[49] Danaei M., Dehghankhold M., Ataei S., Hasanzadeh Davarani F., Javanmard R., Dokhani A., Khorasani S., Mozafari M.R. (2018). Impact of Particle Size and Polydispersity Index on the Clinical Applications of Lipidic Nanocarrier Systems. Pharmaceutics.

[50] Worldwide M.I. (2011). Dynamic Light Scattering, Common Terms Defined.

[51] Bunjes H. (2011). Current Opinion in Colloid & Interface Science Structural properties of solid lipid based colloidal drug delivery systems. Curr. Opin. Colloid Interface Sci..

[52] Zheng M., Falkeborg M., Zheng Y., Yang T., Xu X. (2013). Formulation and characterization of nanostructured lipid carriers containing a mixed lipids core. Colloids Surf. A Physicochem. Eng. Asp..

[53] Puri A., Loomis K., Smith B., Lee J.-H., Yavlovich A., Heldman E., Blumenthal R. (2009). Lipid-based nanoparticles as pharmaceutical drug carriers: From concepts to clinic. Crit. Rev. Ther. Drug Carr. Syst..

[54] Doktorovova S., Souto E.B., Silva A.M. (2014). Nanotoxicology applied to solid lipid nanoparticles and nanostructured lipid carriers—A systematic review of in vitro data. Eur. J. Pharm. Biopharm..

[55] Doktorovová S., Kovačević A.B., Garcia M.L., Souto E.B. (2016). Preclinical safety of solid lipid nanoparticles and nanostructured lipid carriers: Current evidence from in vitro and in vivo evaluation. Eur. J. Pharm. Biopharm..

[56] Ferreira L.E., Muniz B.V., Burga-Sánchez J., Volpato M.C., de Paula E., Rosa E.A., Groppo F.C. (2017). The effect of two drug delivery systems in ropivacaine cytotoxicity and cytokine release by human keratinocytes and fibroblasts. J. Pharm. Pharmacol..

[57] ISO ISO 10993-5:2009: Biological Evaluation of Medical Devices—Part 5: Tests for In Vitro Cytotoxicity. https://www.iso.org/standard/36406.html.

[58] Ghodrati M., Farahpour M.R., Hamishehkar H. (2019). Encapsulation of Peppermint essential oil in nanostructured lipid carriers: In-vitro antibacterial activity and accelerative effect on infected wound healing. Colloids Surf. A Physicochem. Eng. Asp..

[59] Quan C., Cho M.K., Shao Y., Mianecki L.E., Liao E., Perry D., Quan T. (2015). Dermal fibroblast expression of stromal cell-derived factor-1 (SDF-1) promotes epidermal keratinocyte proliferation in normal and diseased skin. Protein Cell.

[60] Iwig M., Glaesser D., Fass U., Struck H.G. (2004). Fatty acid cytotoxicity to human lens epithelial cells. Exp. Eye Res..

[61] Behzadi S., Serpooshan V., Tao W., Hamaly M.A., Alkawareek M.Y., Dreaden E.C., Brown D., Alkilany A.M., Farokhzad O.C., Mahmoudi M. (2017). Cellular uptake of nanoparticles: Journey inside the cell. Chem. Soc. Rev..

[62] Brandelli A. (2020). The interaction of nanostructured antimicrobials with biological systems: Cellular uptake, trafficking and potential toxicity. Food Sci. Hum. Wellness.

[63] Silva E., Barreiros L., Segundo M.A., Costa Lima S.A., Reis S. (2017). Cellular interactions of a lipid-based nanocarrier model with human keratinocytes: Unravelling transport mechanisms. Acta Biomater..

[64] Teskac K., Kristl J. (2010). The evidence for solid lipid nanoparticles mediated cell uptake of resveratrol. Int. J. Pharm..

[65] Rajendran L., Knölker H.J., Simons K. (2010). Subcellular targeting strategies for drug design and delivery. Nat. Rev. Drug Discov..

[66] Neves A.R., Queiroz J.F., Costa Lima S.A., Figueiredo F., Fernandes R., Reis S. (2016). Cellular uptake and transcytosis of lipid-based nanoparticles across the intestinal barrier: Relevance for oral drug delivery. J. Colloid Interface Sci..

[67] Da Silva J.S., Zilly A., da Silva R.M.M., Librelotto C.S., Ferreira H. (2021). Evaluation of Antibacterial Activity of Sunflower Oil: Support for Nursing. Res. Soc. Dev..

[68] Lacey R.W., Lord V.L. (1981). Sensitivity of staphylococci to fatty acids: Novel inactivation of linolenic acid by serum. J. Med. Microbiol..

[69] Lukowski G., Lindequist U., Mundt S., Kramer A., Jülich W.-D. (2008). Inhibition of Dermal MRSA Colonization by Microalgal Micro-and Nanoparticles. Skin Pharmacol. Physiol..

[70] Tabassum N., Vidyasagar G.M. (2014). In vitro antimicrobial activity of edible oils against human pathogens causing skin infections. Int. J. Pharm. Sci. Res. IJPSR.

[71] Kisich K.O., Howell M.D., Boguniewicz M., Heizer H.R., Watson N.U., Leung D.Y. (2007). The constitutive capacity of human keratinocytes to kill *Staphylococcus aureus* is dependent on beta-defensin 3. J. Investig. Dermatol..

[72] Esfanjani A.F., Assadpour E., Jafari S.M. (2018). Improving the bioavailability of phenolic compounds by loading them within lipid-based nanocarriers. Trends Food Sci. Technol..

